# Supplementary material for: Burnout among Chinese live streamers: Prevalence and correlates
Source: PLoS One. 2024 May 21;19(5):e0301984. doi: 10.1371/journal.pone.0301984 (PMC11108200; doi:10.1371/journal.pone.0301984)
Supplement: Attachment — Submitted filename: PONE-D-23-18174_Reviewed.pdf [file pone.0301984.s002.pdf]

PLOS ONE

Burnout prevalence and its associated factors among Chinese webcasters

--Manuscript Draft--

**Manuscript Number:**	PONE-D-23-18174
**Article Type:**	Research Article
**Full Title:**	Burnout prevalence and its associated factors among Chinese webcasters
**Short Title:**	Burnout prevalence of Chinese webcasters
**Corresponding Author:**	Ziwei Liu<br>Hunan Normal University<br>Changsha, Hunan CHINA
**Keywords:**	Burnout; Career-related factors; Interpersonal support; Job stress; Webcasters
**Abstract:**	Background<br><br>Webcaster is a young and high-income job, with a low barrier to entry, attracting a large number of people entering, however, huge job stress, job-related health problems, lack of labour and social security are leading to a wide unhealthy employee turnover. Data on the prevalence of burnout in webcaster are very limited. This study aims to investigate the prevalence and correlates of burnout among Chinese webcaster.<br><br>Methods<br><br>A total of 358 webcasters were recruited from 4 companies in Changsha, China. Socio-demographic information, career-related factors, job stress, interpersonal support, and burnout were collected by a self-design questionnaire through online platform.<br><br>Results<br><br>Nearly half (46.09%, 95% CI: 40.90 ~ 51.02%) of the webcasters met the high burnout criteria. In multiple logistic regression analysis, risk factors associated with burnout include lower education (junior high school and below: OR=9.87), cyber violence (OR=6.79), losing fans (OR=4.37), longer live broadcast duration (>8h per day: OR=3.57), negative performance appraisal (OR=2.61), longer resting (OR=2.56), low income (OR=2.41), live broadcasting time at day (OR=2.30), higher seniority (OR=2.34), job stress (OR=1.19). The protective factor is interpersonal support (OR=0.83).<br><br>Conclusions<br><br>Burnout is prevalent in Chinese webcasters. Dynamic risk factors include losing fans, longer live broadcast duration, negative performance appraisal, low income and daily live broadcast time, as well as job stress. Static risk factors include lower education, cyber violence, and higher seniority. The protective factor is interpersonal support. these findings may contribute to the risk management and health promotion of webcaster’s burnout.
**Order of Authors:**	Shi Chen
	Hanqin Wang
	Shang Yang
	Fushen Zhang
	Xiao Gao
	Ziwei Liu
**Additional Information:**	
**Question**	**Response**

Powered by Editorial Manager® and ProduXion Manager® from Aries Systems Corporation

**Financial Disclosure**<br><br>Enter a financial disclosure statement that describes the sources of funding for the work included in this submission. Review the submission guidelines for detailed requirements. View published research articles from *PLOS ONE* for specific examples.<br><br>This statement is required for submission and **will appear in the published article** if the submission is accepted. Please make sure it is accurate.<br><br>**Unfunded studies**<br>Enter: *The author(s) received no specific funding for this work.*<br><br>**Funded studies**<br>Enter a statement with the following details:<br>• Initials of the authors who received each award<br>• Grant numbers awarded to each author<br>• The full name of each funder<br>• URL of each funder website<br>• Did the sponsors or funders play any role in the study design, data collection and analysis, decision to publish, or preparation of the manuscript?<br>• **NO** - Include this sentence at the end of your statement: *The funders had no role in study design, data collection and analysis, decision to publish, or preparation of the manuscript.*<br>• **YES** - Specify the role(s) played.<br><br>* typeset	This work was supported by the Hunan Normal University undergraduates innovative experiment project and entrepreneurship program (2019116), Hunan Normal University major event social stability risk assessment center 2021-year program (2021WP08).
**Competing Interests**<br><br>Use the instructions below to enter a competing interest statement for this submission. On behalf of all authors, disclose any competing interests that could be perceived to bias this work—acknowledging all financial support and any other relevant financial or non-financial competing interests.<br><br>This statement is required for submission	The authors have declared that no competing interests exist.

Powered by Editorial Manager® and ProduXion Manager® from Aries Systems Corporation

and **will appear in the published article** if the submission is accepted. Please make sure it is accurate and that any funding sources listed in your Funding Information later in the submission form are also declared in your Financial Disclosure statement.<br><br>View published research articles from *PLOS ONE* for specific examples.<br><br>**NO authors have competing interests**<br><br>Enter: *The authors have declared that no competing interests exist.*<br><br>**Authors with competing interests**<br><br>Enter competing interest details beginning with this statement:<br><br>*I have read the journal's policy and the authors of this manuscript have the following competing interests: [insert competing interests here]*<br><br>* typeset	
**Ethics Statement**<br><br>Enter an ethics statement for this submission. This statement is required if the study involved:<br><br>• Human participants<br>• Human specimens or tissue<br>• Vertebrate animals or cephalopods<br>• Vertebrate embryos or tissues<br>• Field research<br><br>Write "N/A" if the submission does not require an ethics statement.<br><br>General guidance is provided below. Consult the submission guidelines for detailed instructions. **Make sure that all information entered here is included in the Methods section of the manuscript.**	Informed consent was obtained from all the study participants. All the procedures of this study were approved by the Medical Ethics Committee of Hunan Normal University (permit number: 2021-283). All methods were carried out in accordance with relevant guidelines and regulations or Declaration of Helsinki.

Powered by Editorial Manager® and ProduXion Manager® from Aries Systems Corporation

**Format for specific study types**<br><br>**Human Subject Research (involving human participants and/or tissue)**<br>• Give the name of the institutional review board or ethics committee that approved the study<br>• Include the approval number and/or a statement indicating approval of this research<br>• Indicate the form of consent obtained (written/oral) or the reason that consent was not obtained (e.g. the data were analyzed anonymously)<br><br>**Animal Research (involving vertebrate animals, embryos or tissues)**<br>• Provide the name of the Institutional Animal Care and Use Committee (IACUC) or other relevant ethics board that reviewed the study protocol, and indicate whether they approved this research or granted a formal waiver of ethical approval<br>• Include an approval number if one was obtained<br>• If the study involved *non-human primates*, add *additional details* about animal welfare and steps taken to ameliorate suffering<br>• If anesthesia, euthanasia, or any kind of animal sacrifice is part of the study, include briefly which substances and/or methods were applied<br><br>**Field Research**<br><br>Include the following details if this study involves the collection of plant, animal, or other materials from a natural setting:<br>• Field permit number<br>• Name of the institution or relevant body that granted permission	
**Data Availability**<br><br>Authors are required to make all data underlying the findings described fully available, without restriction, and from the time of publication. PLOS allows rare exceptions to address legal and ethical concerns. See the PLOS Data Policy and FAQ for detailed information.	No - some restrictions will apply

A Data Availability Statement describing where the data can be found is required at submission. Your answers to this question constitute the Data Availability Statement and **will be published in the article**, if accepted.<br><br>**Important:** Stating 'data available on request from the author' is not sufficient. If your data are only available upon request, select 'No' for the first question and explain your exceptional situation in the text box.<br><br>Do the authors confirm that all data underlying the findings described in their manuscript are fully available without restriction?	
**Describe where the data may be found in full sentences. If you are copying our sample text, replace any instances of XXX with the appropriate details.**<br><br>• If the data are **held or will be held in a public repository**, include URLs, accession numbers or DOIs. If this information will only be available after acceptance, indicate this by ticking the box below. For example: *All XXX files are available from the XXX database (accession number(s) XXX, XXX.).*<br>• If the data are all contained **within the manuscript and/or Supporting Information files**, enter the following: *All relevant data are within the manuscript and its Supporting Information files.*<br>• If neither of these applies but you are able to provide **details of access elsewhere**, with or without limitations, please do so. For example:<br><br>*Data cannot be shared publicly because of [XXX]. Data are available from the XXX Institutional Data Access / Ethics Committee (contact via XXX) for researchers who meet the criteria for access to confidential data.*<br><br>*The data underlying the results presented in the study are available from (include the name of the third party*	Due to the sensitive nature of the questions asked in this study, survey respondents were assured raw data would remain confidential and would not be shared.

and contact information or URL).<br>• This text is appropriate if the data are owned by a third party and authors do not have permission to share the data.<br><br>* typeset	
Additional data availability information:	

Powered by Editorial Manager® and ProduXion Manager® from Aries Systems Corporation

Manuscript Click here to access/download;Manuscript;manuscript.docx

Burnout prevalence and its associated factors among Chinese webcasters

Shi Chen[1,2], Hanqin Wang[2], Shang Yang[2], Fushen Zhang[2], Xiao Gao[2], Ziwei Liu[2 *]

1 National Clinical Research Center for Mental Disorders, Department of Psychiatry, The Second Xiangya Hospital of Central South University, Changsha, China

2 School of medicine, Hunan Normal University, Changsha 410013, China

* Correspondence to: Ziwei Liu, School of Medicine, Hunan Normal University, 371 Tongzipo Road, 410013, Changsha, China. e-mail: liuziwei@hunnu.edu.cn

Abstract

Background: Webcaster is a young and high-income job, with a low barrier to entry, attracting a large number of people entering, however, huge job stress, job-related health problems, lack of labour and social security are leading to a wide unhealthy employee turnover. Data on the prevalence of burnout in webcaster are very limited. This study aims to investigate the prevalence and correlates of burnout among Chinese webcaster.

Methods: A total of 358 webcasters were recruited from three companies in Changsha, China. Socio-demographic information, career-related factors, job stress, interpersonal support, and burnout were collected by a self-design questionnaire through online platform.

Results: Nearly half (46.09%, 95% CI: 40.90 ~ 51.02%) of the webcasters met the high burnout criteria. In multiple logistic regression analysis, risk factors associated with burnout include lower education (junior high school and below: OR=9.87), cyber violence (OR=6.79), losing fans (OR=4.37), longer live broadcast duration (>8h per day: OR=3.57), negative performance appraisal (OR=2.61), longer resting (OR=2.56), low income (OR=2.41), live broadcasting time at day (OR=2.30), higher seniority (OR=2.34), job stress (OR=1.19). The protective factor is interpersonal support (OR=0.83).

Conclusions: Burnout is prevalent in Chinese webcasters. Dynamic risk factors include losing fans, longer live broadcast duration, negative performance appraisal, low income and daily live broadcast time, as well as job stress. Static risk factors include lower education, cyber violence, and higher seniority. The protective factor is interpersonal support. these findings may contribute to the risk management and health promotion of webcaster's burnout.

Keywords: Burnout; Career-related factors; Interpersonal support; Job stress; Webcasters.

Background

Webcaster is defined as a person who broadcasts live over the internet. The rapidly increasing webcasting industry embodies a great economic value. The total amount of financing and the market size related to the webcasting industry have reached 6.23 billion and 193.03 billion, respectively (China Internet Network Information Center, 2020). The webcasting industry brings a huge number of job opportunities and webcaster as a high-income job attracts a large number of people entering. A survey about the income of webcaster in China shows that 93% of webcasters have a monthly income above 4500 Chinese Yuan (CNY), equal to 706 dollars, which is much higher than 2682 CNY of the national per capita income (IiMedia Data Center, 2020). Meanwhile, this job has a low barrier to entry. On one hand, with the optimization and upgrading of the internet communication technology, smartphone and applications, as well as the reduction of holding and use cost, ordinary people have the basic hardware and equipment to be a webcaster. On the other hand, being a webcaster requires almost no special skill training requirement. Hence, the webcaster has developed into an attractive and hot career. By the end of 2020 year, the number of webcasters accounts has reached 130 million in China and new accounts are added to over 43 thousand every day (China Internet Network Information Center, 2020).

Webcaster is a young job. Compared with the developed webcasting operational system, its occupational safety and health administration is still in its infancy. Webcasters are undertaking an excessiveness of working time or burden and a disturbance of working rhythm. Furthermore, some unique working features like keeping

a fixed working posture, rapid decrement of fans amount, abuse from the audience in the broadcast site through the barrage, and failure for work performance appraisal from companies, etc. Unreasonable work arrangements produce a big work pressure and job stress. Long-term overload job stress further leads to a series of health problems. However, the vast majority of webcasters have not received occupational health support except basic medical insurance, or even without any medical insurance from the company. As a result, huge job stress, job-related health problems, lack of labor and social security are leading to a wide unhealthy employee turnover in webcasting companies.

Burnout is a state of physical and mental fatigue of laborers within the service industry due to long-term, high-intensity, and high-load work (Bianchi et al., 2015), characterized by emotional exhaustion, cynicism, and negative self-evaluation (Maslach et al., 2001). Burnout is one of the major outcomes of long-term unresolved job stress, which directly reduces the efficiency and quality of work but as well as harms physical and mental health, for instance, depression or insomnia (Jaegers et al., 2021; Sygit-Kowalkowska et al., 2021). Hence, burnout is not only an indicator of overload burden but also one crucial warning sign of health hazards. Performing intervention before burnout occurs can effectively prevent occupational injuries. However, data on the prevalence of burnout in webcasters are very limited. This leads to insufficient evidence for identifying and early warning burnout among the webcaster population.

How to make job of webcaster become a sustainable long-term career? Job stress, job-related health problems, and deficient labor and health security regimes increase the occupational hazards and shorten the career longevity. Therefore, this study aims to investigate the prevalence and correlates of burnout among Chinese webcasters to provide evidence for making occupational health and safety policies in the webcasting industry.

Methods

Ethical approval and consent to participants

This study was a descriptive cross-sectional survey conducted in three broadcasting companies of Changsha, Hunan. This survey was conducted by WWW.WJX.CN and WENJUAN.COM, which were two secure electronic survey platforms in mainland China. All the procedures of this study were approved by the Medical Ethics Committee of Hunan Normal University (permit number: 2021-283). All participants accepted voluntary participation and obtained electronic informed consent. Informed consents of participants aged 16 to 18 years obtained from their guardians.

Participants

This study was a descriptive cross-sectional survey. Participants were recruited via a random cluster sampling from broadcasting companies of Changsha, Hunan, between Oct. 2021 and Nov. 2021. Inclusion criteria: 1) the full-time webcaster; 2) over 16 years old; 3) a contract was signed with a webcasting company. Exclusion criteria: 1) the part-time webcasters who have not signed a contract with a webcasting company; 2) infection of certain nervous system related diseases or mental disorders affected their communication function, for instance, general anxiety disorder, major depressive disorder, etc.

A total of 369 webcasters were recruited from four broadcasting companies to participate in the study. Of which, 11 had missing values on variables and were excluded from the present study resulted in the quailed ratio of 97%. Finally, 358 participants were included in our sample.

Measures

Dependent variable

Burnout. The burnout levels of webcasters were evaluated in the revised Chinese version of the Maslach burnout inventory-human services survey (MBI-HSS) (Zhang et al., 2006). Each item was rated on a 6-point score, ranging from 0 (none of the time) to 6 (every day). MBI-HSS is characterized by three dimensions, namely, 7-item emotional exhaustion (EE), 3-item depersonalization (DP), and 7-item personal accomplishment (PA). We set the mean of each dimension as a critical value. Further, both high-EE and high-DP (above average) were judged as the group with high-burnout (Leigh et al., 2020). Such a criterion has been validated in many studies (Maslach et al., 1981; Rotenstein et al., 2018; Zhang et al., 2021). This study divided Chinese webcasters into a high-burnout group and low-burnout group for follow-up analysis. The Cronbach's alpha coefficients for the MBI-HSS, EE, DP, and PA were 0.96, 0.95, 0.90, and 0.94, respectively, suggesting that the overall measurement was reliable.

Independent variables

Socio-demographic characteristics. Age, gender, education (junior high school and below/high, vocational high, or technical secondary school/junior college/undergraduate), and income were collected.

Career-related factors. Seniority (less than 1 year/more than 1 year), the type of live stream (entertainment webcaster/game webcaster/other), work posture (prolonged standing/prolonged sitting/prolonged walking/no fixed position), the change of fans amount (a great increasement/a slight increasement/no obvious change/an obvious decrement), days for resting per month (less than 4 days/ more than 4 days), live broadcast duration (2-4h/4-6h/6-8h/>8h, preliminary investigations suggested that Chinese webcaster's daily live broadcast duration are at least 2h), live broadcast time (mostly at day/mostly at night), work performance appraisal (positive/negative), cyber violence experiment (Respondents need to answer the question "Have you experienced cyber violence": yes/no).

Job stress. Job stress is conceptualized as an imbalance of an individual's reaction between the individual's abilities and the work environment (Jamal et al., 2000). The scores of job stress of Chinese webcasters were measured by the Job Content Questionnaire (JCQ-22). It was a 22-item self-report scale. Each item was rated on a 4-point score, ranging from 1 (strongly disagree) to 4 (strongly agree). It was used to measure job demands (5 items), job control (9 items), and social support (i.e., supervisor and coworker support, 8 items), respectively (Ikeda et al., 2021). A high total score of job demands and job control suggested a serious level of job stress (Jiang et al., 2019). The Cronbach's alpha coefficients for the JCQ-22, job demands and job control were 0.94, 0.90, and 0.95, respectively, suggesting that the overall measurement was reliable.

Interpersonal support. Interpersonal support refers to some material or spiritual assistance obtained without compensation from social circle. The scores of interpersonal supports of Chinese webcasters were also measured by JCQ-22. It was used to measure job demands (5 items), job control (9 items), and social support (i.e., supervisor and coworker support, 8 items), respectively (Ikeda et al., 2021). A high score of social support

meant a stronger level of interpersonal support (Jiang et al., 2019). The Cronbach's alpha coefficients for social support was 0.95.

Covariates

Age and gender are two covariates in our study which were measured in the content of socio-demographics.

Statistical analysis

SPSS software version 22.0 was used to perform statistical analysis. Socio-demographic and career-related factors were described and compared by t-test or Chi-square test. Multiple logistic regression with a back-step entry of significant variables in the above univariate analysis was used to identify factors significantly associated with burnout. Odds ratios (ORs) and 95% confidence intervals (CIs) were used to testify the associations between burnout and factors. The statistical significance level was set at $P < 0.05$ (two-tail).

Results

Participant characteristics

Of the 358 webcasters, 57% of them are female; 68% of them are less than or equal to 25 years old; 38% of them have an educational background of junior college. The detailed socio-demographic characteristics are shown in **Table 1**.

Burnout level of webcaster

As for MBI-HSS score, we found that the average score of emotional exhaustion was 26.29; the average score of depersonalizations was 12.01; the average score of personal accomplishment was 28.24. Then, of the 358 webcasters, 46.09% (165 of 358, 95% CI: 40.90 ~ 51.02%) of them reached high burnout level and 54. 91% (193 of 358) of them were low level burnout.

Univariate diffidence between high- and low-level burnout groups

The detailed socio-demographic characteristic, career-related factors, overall scores of job stress and interpersonal support were shown in **Table 1**. There are differences in burnout levels exist with the following characteristics among Chinese webcasters: educational background (P<0.001), monthly income (P<0.001), seniority (P<0.001), work posture (P=0.017), change of fans amount (P=0.013), the day for resting (P=0.001), live broadcast duration (P<0.001), live broadcast time (P<0.001), work performance appraisal (P=0.010), an experience of cyber violence (P<0.001). Also, significant differences both exist in various scores of job stress or interpersonal support among the population of Chinese webcasters (P<0.001).

Associated factors of burnout among webcasters

Results from multivariable logistic regression (**Table 2**) showed that factors significantly associated with burnout were an educational background of junior college (vs. undergraduate, OR=2.33), high, vocational high, or technical secondary school (vs. undergraduate, OR=3.48), junior high school and below (vs. undergraduate, OR=9.87); an average income of 5000-10000 (vs. > 10000, OR=2.41); seniority of more than 1 year (vs. seniority of less than 1 year, OR=2.34), an obvious decrement of fans amount (vs. a great increasement of fans amount, OR=4.37); a more than 4-day for resting (vs. a less than 4-day for resting OR=2.56); a 4-6h-daily live

broadcast duration (vs. 2-4h-daily live broadcast duration, OR=2.81), a 6-8h-daily live broadcast duration (vs. 2-4h-daily live broadcast duration, OR=3.59), a >8h-daily live broadcast duration (vs. 2-4h-daily live broadcast duration, OR=3.57); a daily live broadcast time at night mostly (vs. mostly at day, OR=2.30); negative performance appraisal (vs. positive performance appraisal, OR=2.61); an ever cyber violence experience (vs. no experience of cyber violence, OR=6.79). Also, the difference was significant in job stress scores (OR=1.19) and interpersonal support scores between the two groups of "high-burnout" and "low-burnout" (OR=0.83).

Discussion

Main findings

Nearly half (46.09%, 95% CI: 40.90 ~ 51.02%) of the webcasters met the high burnout criteria. In multiple logistic regression analysis, risk factors associated with burnout include lower education (junior high school and below: OR=9.87), cyber violence (OR=6.79), losing fans (OR=4.37), longer live broadcast duration (>8h per day: OR=3.57), negative performance appraisal (OR=2.61), longer resting (OR=2.56), lower income (OR=2.41), broadcasting time at day (OR=2.30), higher seniority (OR=2.34), job stress (OR=1.19). The protective factor is interpersonal support (OR=0.83).

We divided these associated factors into three categories: dynamic risk factor (losing fans, a longer live broadcast duration, negative performance appraisal, daily live broadcast time, and job stress), static risk factor (education, cyber violence, and seniority), and protective factor (interpersonal support).

Dynamic risk factors

Dynamic risk factors are changeable and provide the opportunity for intervention. Out study finds the dynamic risk factors include losing fans, a longer live broadcast duration, negative performance appraisal, and daily live broadcast time, as well as job stress. Among these factors, almost all are career-related factors except losing fans. An obvious decrement of fans' amount is a strong risk factor for burnout. Losing fans indicates a webcaster makes a mistake at work and it closely associates with work performance appraisal.

Career-related factors are the most concerned and discussed in detail. Compared to webcasters who have a live broadcast at night mostly, having a live broadcast at day mostly was a dangerous factor for Chinese webcasters' burnout, indicating that disturbance of circadian rhythm might be one of the important reasons for Chinese webcasters' burnout (Canadas-De et al., 2015). Excessive live broadcast duration and negative performance appraisal might also aggravate the burnout level of Chinese webcasters, which also indicates the working environment of the webcaster is still immature. Unreasonable work arrangements will result in a reduced work efficiency and some potential occupational hazard and health problem. Through scientific and reasonable planning and arrangements, work pressure and job stress can be alleviated. Hence, establishing a monitoring and control system towards dynamic risk factors will be beneficial for early warning of burnout among webcaster.

Static risk factors

Static risk factors are historical and do not change or passive and not easy to change, which indicates that it is difficult to intervene these factors to reduce the risk of burnout. Out study finds the static risk factors include

education, cyber violence, and seniority. Webcaster with an education background of high school and below shows the highest risk of burnout. Worries resulted from low education are very common in many kinds of careers. However, lower education is the biggest risk factor of burnout in our study. This is an interesting phenomenon. Because when webcasters are hired by companies, they were not requiring a high education and which is also not deciding the webcaster income. We think the possible reason is that educational background may play a big role in peer competition and gain promotion in the course of work. Following education, cyber violence is the second biggest risk factor of burnout. Being bullied verbally online is quite common on online social media (Chen et al., 2018; France et al., 2013; Taein et al., 2020). Our study finds that cyber violence is a strong predictor of burnout in Chinese webcasters. For webcaster, cyber violence is difficult to avoid and there is no effective tools, approach, or mechanism to assess and copy with the injures resulted from cyber violence. We think it is necessary to provide regular training on cyber violence response methods, and provide regular psychological examination, as well as mental health services for webcaster. Seniority changes with the working years. But for individual employee, seniority is non-voluntary factor. In the present study, webcasters with more than 1 year of seniority have a higher risk of burnout. We speculate those who has longer seniority might appear poor creativity gradually and cause a decline in interaction or rewards during live broadcasts, thereby leading to burnout. Anyway, it is impossible to reduce the burnout risk through changing static risk factors directly. But this also reminds us that we could use classification management and different kinds of support approaches to control the risk of burnout among webcaster with different risk factors.

Protective factor

Interpersonal support is unlikely to directly cause burnout. A worse interpersonal support can intensify the bad effect of risk factors. However, a good interpersonal support can play a "buffer" effect between job stress and adverse outcomes including burnout. Good interpersonal support helps to reduce the individual traumatic feeling and assessment towards injury of job stress, and increase the self-efficiency and coping ability.

Limitations

First, our statistical model of burnout risk factors has implications for assessing the scale of risk on individual level. However, it needs to be noted there are individual differences in the types and contributions of risk factors between each other. Second, as there is no consensus on the diagnosis of burnout, it is difficult to compare the prevalence of burnout directly. A recent review found that the existing literature used at least 47 different definitions of the prevalence of burnout when using the MBI tool to measure burnout (Rotenstein et al., 2020). Therefore, future studies need to reach a consensus on how to classify different degrees of burnout, thereby comparing with that of other occupations. Finally, we only described a basic situation of burnout but not discussed its three dimensions (namely EE, DP, and PA) in depth. In future research, the relationship between them and specific career-related factors of Chinese webcasters still need to be further analyzed.

Conclusion

Burnout is prevalent in Chinese webcasters. Correlates of webcasters' burnout can be classified into dynamic and static risk factors as well as protective factors. Dynamic risk factors include losing fans, longer live broadcast duration, negative performance appraisal, daily live broadcast time, lower income and job stress. Static risk factors include higher seniority, lower education, and cyber violence. The protective factor is

interpersonal support. These findings above proposed some new ideas that can be further applied to explore an occupational safety and health administration of Chinese webcasters.

Declarations

Ethical approval and consent to participants

All methods were carried out in accordance with relevant guidelines and regulations or Declaration of Helsinki. All the procedures of this study were approved by the Medical Ethics Committee of Hunan Normal University (permit number: 2021-283). All participants accepted voluntary participation and obtained electronic informed consent. Informed consents of participants aged 16 to 18 years obtained from their guardians.

Consent for publication

Not applicable.

Availability of data and materials

The datasets generated and analysed during the current study are not publicly available due containing the private information of the participants but are available from the corresponding author on reasonable request.

Competing interests

The authors declare no conflict of interest.

Authors' contributions

Shi Chen and Ziwei Liu contributed equally to the study design and research work. Hanqin Wang and Shang Yang have conducted the survey and wrote the first-draft manuscript. Fushen Zhang have revised it. Xiao Gao finished the statistical analysis.

Funding

This work was supported by the Hunan Normal University undergraduates innovative experiment project and entrepreneurship program (2019116), Hunan Normal University major event social stability risk assessment center 2021-year program (2021WP08).

Acknowledgements

The authors would like to acknowledge the staff from wan hui media co., LTD of Changsha.

Reference

Internet Network China Information Center. The 47th China Statistical Report on Internet Development. 2020: http://www.cac.gov.cn/2021-02/03/c_1613923423079314.htm. Accessed 14 July 2021.

IiMedia Data Center. Monthly profit distribution of Chinese webcasters in 2020H1.2020: https://data.iimedia.cn/page-category.jsp?nodeid=30412956. Accessed 17 November 2021.

Bianchi, R., Schonfeld, I. S., & Laurent, E. Burnout-depression overlap: a review. Clinical psychology review. 2015;36, 28–41. https://doi.org/10.1016/j.cpr.2015.01.004

Maslach, C., Schaufeli, W. B., & Leiter, M. P. Job burnout. Annual review of psychology. 2001; 52, 397–422. https://doi.org/10.1146/annurev.psych.52.1.397.

Jaegers, L. A., Vaughn, M. G., Werth, P., Matthieu, M. M., Ahmad, S. O., & Barnidge, E. Work-Family Conflict, Depression, and Burnout Among Jail Correctional Officers: A 1-Year Prospective Study. Safety and health at work. 2021;12(2), 167–173. https://doi.org/10.1016/j.shaw.2020.10.008.

Sygit-Kowalkowska, E., Piotrowski, A., & Hamzah, I. Insomnia among Prison Officers and Its Relationship with Occupational Burnout: The Role of Coping with Stress in Polish and Indonesian Samples. International journal of environmental research and public health. 2021; 18(8), 4282. https://doi.org/10.3390/ijerph18084282.

Zhang, SY. and Yan, X., 2006. Revision and application of MBI-Human services survey in Chinese police officers. Chinese Mental Health Journal.

Leigh, R., Van Aarsen, K., Foxcroft, L., & Lim, R. P012: Does physician burnout differ between urban and rural emergency medicine physicians? A comparison using the Maslach Burnout Inventory tool. CJEM. 2020; 22(S1), S68-S69. doi:10.1017/cem.2020.220

Maslach, C. and Jackson, S.E. The measurement of experienced burnout. J. Organiz. Behav. 1981; 2: 99-113. https://doi.org/10.1002/job.4030020205.

Rotenstein, L. S., Torre, M., Ramos, M. A., Rosales, R. C., Guille, C., Sen, S., & Mata, D. A. Prevalence of Burnout Among Physicians: A Systematic Review. JAMA. 2018; 320(11), 1131–1150. https://doi.org/10.1001/jama.2018.12777.

Zhang, X., Wang, J., Hao, Y., Wu, K., Jiao, M., Liang, L., Gao, L., Ning, N., Kang, Z., Shan, L., He, W., Wang, Y., Wu, Q., & Yin, W. Prevalence and Factors Associated With Burnout of Frontline Healthcare Workers in Fighting Against the COVID-19 Pandemic: Evidence From China. Frontiers in psychology. 2021;12, 680614. https://doi.org/10.3389/fpsyg.2021.680614.

Jamal, M., & Baba, V. V.   Job stress and burnout among Canadian managers and nurses: an empirical examination. Canadian journal of public health = Revue canadienne de sante publique.2000;91(6), 454–458. https://doi.org/10.1007/BF03404828.

Ikeda, S., Eguchi, H., Hiro, H., Mafune, K., Koga, K., Nishimura, K., & Nakashima, M. Work-Family Spillover, Job Demand, Job Control, and Workplace Social Support Affect the Mental Health of Home-Visit Nursing Staff. Journal of UOEH. 2021;43(1), 51–60. https://doi.org/10.7888/juoeh.43.51.

Jiang, XW., Ju, W., Chang, C. Path analysis of anxiety and depression status and its work environment related among occupational population. Chinese Mental Health Journal. 2019; 05, 375-380.

Cañadas-De la Fuente, G. A., Vargas, C., San Luis, C., García, I., Cañadas, G. R., & De la Fuente, E. I. Risk factors and prevalence of burnout syndrome in the nursing profession. International journal of nursing studies. 2015;52(1), 240–249. https://doi.org/10.1016/j.ijnurstu.2014.07.001.

Chen, Q., Chan, K. L., & Cheung, A. Doxing Victimization and Emotional Problems among Secondary School Students in Hong Kong. International journal of environmental research and public health. 2018;15(12), 2665. https://doi.org/10.3390/ijerph15122665.

France, K. , Danesh, A. , & Jirard, S. Informing aggression–prevention efforts by comparing perpetrators of brief vs. extended cyber aggression. Computers in Human Behavior. 2013;29(6), 2143-2149.

Eom, T., & Lim, C. Interactive Art that informs the seriousness of cyber verbal violence - “Blame.” International Journal of Advanced Culture Technology. 2020;8(1), 188–198. https://doi.org/10.17703/IJACT.2020.8.1.188.

Table 1. Characteristics of participants and prevalence rate of burnout by variables

Variables	Total	High-burnout	Rate of high-burnout (%)	χ2/t	*P*
N	358	165	46.09		
**Gender**					
Male	153	64	41.83	1.95	0.162
Female	205	101	49.27		
**Age**					
16 to 25 years old	242	125	51.65	0.26	0.611
More than 25 years old	116	40	34.48		
**Educational background**					
Undergraduate	103	32	31.07	22.13	＜0.001
Junior college	137	62	45.26		
High, vocational high, or technical secondary school	76	41	53.95		
Junior high school and below	42	30	71.43		
**Monthly income (CNY)**					
> 10000	106	48	45.28	17.76	＜0.001
5000 ~ 10000	176	97	55.11		
< 5000	76	20	26.32		
**Seniority**					
Less than 1 year	92	26	28.26	15.84	＜0.001
More than 1 year	266	139	52.26		
**The type of live stream**					
Entertainment webcaster	141	71	50.35	2.45	0.293
Game webcaster	122	56	45.90		
Other	95	38	40.00		
**Work posture**					
Prolonged walking	51	24	47.06	10.25	0.017
Prolonged standing	172	82	47.67		
Prolonged sitting	64	37	57.81		
No fixed position	71	22	30.99		
**Change of fans amount**					
A great increasement	47	15	31.91	10.85	0.013
A slight increasement	58	23	39.66		

No obvious changes	134	59	44.03		
An obvious decrement	119	68	57.14		
**Days for resting per month**					
Less than 4 days	235	94	40.00	10.21	0.001
More than 4 days	123	71	57.72		
**Daily live broadcast duration**					
2-4h	76	22	28.95		
4-6h	120	50	41.67	18.43	＜0.001
6-8h	122	69	56.56		
>8h	40	24	60.00		
**Daily live broadcast time**					
Mostly at night	112	36	32.14	12.76	＜0.001
Mostly at day	246	129	52.44		
**Work performance appraisal**					
Positive	134	50	37.31	6.64	0.010
Negative	224	115	51.34		
**Cyber violence**					
No	123	22	17.89	59.99	＜0.001
Yes	235	143	60.85		
**Job stress**	38.95±10.52 [a]	-	-	-6.553	＜0.001
**Interpersonal support**	22.60±6.05 [a]	-	-	5.537	＜0.001

a indicate Mean±SD.

Table 2. Associated Factors for burnout among Chinese webcasters

Variables	Univariate		Multivariate	
	OR (95% CI)	*P*	OR (95% CI)	*P*
**Dynamic risk factors**				
**Educational background**				
Undergraduate	1		1	
Junior college	1.83 (1.07, 3.14)	0.040	2.33 (1.11, 4.88)	0.044
High, vocational high, or technical secondary school	2.60 (1.41, 4.81)	0.004	3.48 (1.50, 8.08)	0.002
Junior high school and below	5.55 (2.52, 12.21)	＜0.001	9.87 (3.33, 29.28)	＜0.001
**Monthly income (CNY)**				
> 10000	1		1	
5000-10000	1.48 (0.91, 2.41)	0.064	2.41 (1.23, 4.71)	0.003
< 5000	0.43 (0.23, 0.82)	0.013	0.63 (0.27, 1.49)	0.251
**Seniority**				
Less than 1 year	1		1	
More than 1 year	2.78 (1.66, 4.64)	＜0.001	2.34 (1.09, 5.00)	0.017
**Change of fans amount**				
A great increasement	1		1	
A slight increasement	1.40 (0.63, 3.15)	0.413	0.68 (0.22, 2.14)	0.537
No obvious changes	1.68 (0.83, 3.39)	0.148	1.47 (0.58, 3.74)	0.071
An obvious decrement	2.84 (1.40, 5.80)	0.004	4.37 (1.64, 11.66)	0.010
**Days for resting per month**				
Less than 4 days	1		1	
More than 4 days	2.05 (1.32, 3.19)	0.002	2.56 (1.37, 4.79)	0.001
**Daily live broadcast duration**				
2-4h	1		1	
4-6h	1.75 (0.95, 3.24)	0.073	2.81 (1.16, 6.77)	0.024
6-8h	3.20 (1.73, 5.89)	＜0.001	3.59 (1.49, 8.63)	0.007
>8h	3.68 (1.65, 8.22)	0.001	3.57 (1.20, 10.60)	0.037
**Daily live broadcast time**				
Mostly at night	1		1	
Mostly at day	2.33 (1.46, 3.72)	＜0.001	2.30 (1.20, 4.43)	0.018
**Work performance appraisal**				
Positive	1		1	
Negative	1.77 (1.15, 2.75)	0.014	2.61 (1.39, 4.90)	0.002
**Static risk factors**				

**Cyber violence**				
No	1		1	
Yes	7.14 (4.20, 12.13)	＜0.001	6.79 (3.43, 13.44)	＜0.001
**Job stress scores**	1.07 (1.05, 1.10)	＜0.001	1.19 (1.11, 1.27)	＜0.001
**Protective factor**				
**Interpersonal support scores**	1.07 (1.04, 1.12)	＜0.001	0.83 (0.74, 0.93)	0.001

raw data

Click here to access/download
Supporting Information
rawdata.sav

Original document of ethics committee

Click here to access/download
Supporting Information
WechatIMG1447.jpeg

Supporting Information

Click here to access/download
Supporting Information
EnglishTranlation.docx
