# Supplementary material for: Burnout among Chinese live streamers: Prevalence and correlates
Source: PLoS One. 2024 May 21;19(5):e0301984. doi: 10.1371/journal.pone.0301984 (PMC11108200; doi:10.1371/journal.pone.0301984)
Supplement: Attachment — Submitted filename: Response to Reviewers.docx [file pone.0301984.s003.docx]

**Response to Reviewers**

Dear All,

Thank you very much for your thorough review of our manuscript. During the revision process, we identified some statistical errors, prompting us to reexamine the raw data and conduct all the statistical analyses anew. The updated statistical results significantly differ from the previous ones, leading us to revise the logic and content of the paper accordingly. As a result, it took us longer than anticipated. We kindly request you to review the revised manuscript once again, and we appreciate your efforts and understanding.

Ziwei Liu

Feb.15 2024

Reviewer #1: The paper addresses an important and timely topic since job burnout is pervasive and new risks are emerging in web-based activities.
I do have a few comments, however, on which I would like to ask the authors to respond.
(1) Dependent variable: The classification into groups with high or low burnout scores should be explained in more detail.
- Does &quot;mean of each dimension&quot; refer to the sample mean? And were no external norms used? If so, approximately half of the sample is considered to be at risk, even if the values are, for example, in the lower range compared to other professions. That would make it difficult to determine the actual risk of burnout.

Response：Through literature review, we found that the practice of simply using the total score of the Maslach Burnout Inventory-Human Services Survey (MBI-HSS) to indicate burnout does not adhere to the original scale's guidelines. Therefore, we have redefined the categorization of burnout based on the following two studies.

[7] Rotenstein LS, Torre M, Ramos MA, Rosales RC, Guille C, Sen S, Mata DA. Prevalence of Burnout Among Physicians: A Systematic Review. JAMA. 2018;320(11):1131-1150. doi: 10.1001/jama.2018.12777.

[16] Upton D, Mason V, Doran B, Solowiej K, Shiralkar U, Shiralkar S. The experience of burnout across different surgical specialties in the United Kingdom: a cross-sectional survey. Surgery. 2012;151(4):493-501. doi: 10.1016/j.surg.2011.09.035.

In the revised manuscript, we provided a detailed description as follows:

“…Burnout was defined as a combination of a high score on emotional exhaustion and a high score on depersonalization. High scores in either of the dimensions were defined as scores exceeding the top tentile. In the current study, the Cronbach’s alpha for the MBI-HSS was 0.96. The top tertile of emotional exhaustion corresponded to a score of 27, while a score of 10 represented the threshold for depersonalization. Therefore, individual who scored >27 on the emotional exhaustion ‘and’ scored > 10 on the depersonalization met the criteria for burnout. ”

- &ldquo;Further, both high-EE and high-DP (above average) were judged as the group with high-burnout&rdquo;: does this mean that PA was not considered for the classification?
- Studies were cited which used a similar approach. However, the MBI provides norm-based cutoff values for &quot;low risk,&quot; &quot;moderate risk,&quot; and &quot;high risk.&quot; Why were these not considered?
Response：Thank you for your reminder. We searched the literature again and examined the cutoff values provided by the MBI. But we used the revised Chinese version of the 17-item Maslach Burnout Inventory specifically tailored to the Chinese population, instead of the original 22-item version. However, there is currently no universally accepted cutoff score for this scale. We employed the top tertile as the cutoff, a method used in other study as well [16]. We added this description in the section of discussion and discussed the potential implications of tertile cutoff in our study.

(2) Discussion, Main findings: It is useful to briefly repeat the main results, but the statistical metrics (CI, OR) should not be repeated.

Response: We have made the necessary revisions based on your suggestions. Please review it again.

(3) Discussion, Dynamic risk factors:
- I didn&#39;t entirely understand why a nighttime broadcast is less dangerous than a daytime one. Is night work the norm in this profession?
- Overall, the discussion could make more reference to theory and previous studies.

Response：We re-evaluated the data and do all of the statistical analyses again in the new dataset. We found conducting live stream during daytime remains a significant risk factor compared to evening. The public usually watch livestreams for entertainment purposes. Burnout associated with live streaming during the daytime is likely because the prime time for live streaming is usually in the evening when the public has time to watch. Based on our understanding, for high-performing, popular, and influential streamers, their work is primarily scheduled for the evening. In contrast, streamers with lower performance and influence may be assigned to stream during the daytime. This could be related to their state of burnout, but the specific mechanisms need further investigation. We add the description in the section of discussion in the revised manuscript. And also we found the simple categorization of risk factors into dynamic and static factors was not appropriate, we have abandoned this classification method in the revised paper.

(4) Discussion, Protective factor: &ldquo;Interpersonal support is unlikely to directly cause burnout&rdquo;: However, the analysis model tested direct influence (and it was significant), right? No moderator effects of &quot;interpersonal support&quot; were tested either (if I interpret it correctly). Thus, these conclusions can actually only represent hypotheses for future research.
Reponse：“interpersonal support”in the revised manuscript we used a more accurate description of “supervisor and coworker support”. Because the last 8 items of the JCQ-22 to measure the social support, instead of a professional social support scale. These 8 items consist of four items represent the support from supervisors and remaining four items measuring the support from coworkers.

In our new multivariate logistic model, supervisor and coworker support was found to be a risk factor, rather than a protective factor, which contradicts the findings in the teacher and nurse populations. We conducted more analysis on the results and added relevant descriptions in the discussion section. Please review it again.

(5) Minor comments:
- The p-value is usually written in lower case.
- I am not a native English speaker, but a few phrases seemed to me linguistically not quite correct. I recommend having the text checked again.

Response: We have made the necessary revisions based on your suggestions. Thanks again.

Reviewer #2: (1) How was this set of the exclusion criteria determined? The marked content is “infection of certain nervous system related diseases or mental disorders affected their communication function, for instance, general anxiety disorder, major depressive disorder

Response: we reexamined our study design and execution records. The description was not appropriate. Actually, the investigators assessed whether participants were able to read and respond to the content of the questionnaire. We revised the description as follows: Participants were excluded if they: (1) had not signed a contract with a broadcasting company; (2) were younger than 16; (3) had difficulties in reading or communicating; and (4) were unable to independently complete questionnaires using electronic devices.

(2) Provide information on the total score per dimension and the cut off for each dimension. How was personal accomplishment categorized into high or low burn out?

Response：Through literature review, we found that the practice of simply using the total score of the Maslach Burnout Inventory-Human Services Survey (MBI-HSS) to indicate burnout does not adhere to the original scale's guidelines. Therefore, we have redefined the categorization of burnout based on the following two studies.

[7] Rotenstein LS, Torre M, Ramos MA, Rosales RC, Guille C, Sen S, Mata DA. Prevalence of Burnout Among Physicians: A Systematic Review. JAMA. 2018;320(11):1131-1150. doi: 10.1001/jama.2018.12777.

[16] Upton D, Mason V, Doran B, Solowiej K, Shiralkar U, Shiralkar S. The experience of burnout across different surgical specialties in the United Kingdom: a cross-sectional survey. Surgery. 2012;151(4):493-501. doi: 10.1016/j.surg.2011.09.035.

In the revised manuscript, we provided a detailed description as follows:

“…Burnout was defined as a combination of a high score on emotional exhaustion and a high score on depersonalization. High scores in either of the dimensions were defined as scores exceeding the top tentile. In the current study, the Cronbach’s alpha for the MBI-HSS was 0.96. The top tertile of emotional exhaustion corresponded to a score of 27, while a score of 10 represented the threshold for depersonalization. Therefore, individual who scored >27 on the emotional exhaustion ‘and’ scored > 10 on the depersonalization met the criteria for burnout. ”

(3) Did the authors take 'assent' from the participants who were aged 16-18.

Response：Three participants aged 16 -18 was recruited in this study. We obtained assent from the participants themselves and consent from their guardians before the interview.

(4) Other minor comments

Response: We have made the necessary revisions based on your suggestions. Please review it again.
