# Supplementary material for: Burnout among Chinese live streamers: Prevalence and correlates
Source: PLoS One. 2024 May 21;19(5):e0301984. doi: 10.1371/journal.pone.0301984 (PMC11108200; doi:10.1371/journal.pone.0301984)
Supplement: Attachment — Submitted filename: Response to Reviewers.docx [file pone.0301984.s004.docx]

**Response to Reviewers**

**Reviewer comments:**

I would like to thank the authors for their conscientious revision of the manuscript, taking into account the reviewers' comments.
Most of the points are now clarified to me.

I only have one follow-up question concerning burnout classification:
I understand that the Chinese version has a different number of items and that there are no meaningful threshold values. The procedure with the combined risk as a criterion also seems reasonable to me.
However, the relevance of personal accomplishment is still not clear to me. Although the subscale is mentioned, it is not taken into account for the burnout classification, only emotional exhaustion and depersonalization. Yet, low personal accomplishment would also be an indicator of burnout. Is there a reason why this subscale was not included in the chosen approach?

**Response:** Initially, we used the following criteria to determine burnout：higher scores on the emotional exhaustion and depersonalization subscales and lower scores on the personal accomplishment subscale correspond to higher levels of burnout。[Rotenstein LS, Torre M, Ramos MA, Rosales RC, Guille C, Sen S, Mata DA. Prevalence of Burnout Among Physicians: A Systematic Review. JAMA. 2018 Sep 18;320(11):1131-1150. doi: 10.1001/jama.2018.12777. PMID: 30326495; PMCID: PMC6233645.]

However, the statistical analysis results showed that only 3 of participants met the criteria: EE> 27, DP >10 ‘and’ PA<17 (the bottom tertile among the 343 participants). This clearly appears to be an unreasonable result.

Maslach in his article mentioned that personal accomplishment cannot be assumed to be the opposite of EE and DP. The correlations between the personal accomplishment subscale and the other subscales are low. [Maslach, Christina & Jackson, Susan & Leiter, Michael. (1997). The Maslach Burnout Inventory Manual.] But in our sample, we found that personal accomplishment was not negatively associated with emotional exhaustion and depersonalization but rather positively and significantly associated with them. In other words, individuals with higher levels of emotional exhaustion and depersonalization also exhibited higher levels of personal accomplishment. Despite excluding the possibility of data errors, it remains unclear whether this phenomenon is a unique characteristic of the live streamer population or if it is influenced by other confounding factors. This necessitates further investigation through an independent study to explore the underlying reasons.

Considering the strong correlation between personal accomplishment and emotional exhaustion or depersonalization, we did not use the PA subscale to assist in determining burnout in this study. We used the criteria reported in other studies as follow, which consider only scores higher than 27 on the emotional exhaustion scale ‘and’ scores higher than 10 on the depersonalization scale to determine burnout.

[Pedersen AF, Ingeman ML, Vedsted P. Empathy, burn-out and the use of gut feeling: a cross-sectional survey of Danish general practitioners. BMJ Open. 2018 Feb 28;8(2):e020007. doi: 10.1136/bmjopen-2017-020007. PMID: 29490966; PMCID: PMC5855338.]

[Shanafelt TD, West CP, Sloan JA, Novotny PJ, Poland GA, Menaker R, Rummans TA, Dyrbye LN. Career fit and burnout among academic faculty. Arch Intern Med. 2009 May 25;169(10):990-5. doi: 10.1001/archinternmed.2009.70. PMID: 19468093.]

[Upton D, Mason V, Doran B, Solowiej K, Shiralkar U, Shiralkar S. The experience of burnout across different surgical specialties in the United Kingdom: a cross-sectional survey. Surgery. 2012 Apr;151(4):493-501. doi: 10.1016/j.surg.2011.09.035. Epub 2011 Nov 16. PMID: 22088818.]

[Shanafelt TD, Boone S, Tan L, Dyrbye LN, Sotile W, Satele D, West CP, Sloan J, Oreskovich MR. Burnout and satisfaction with work-life balance among US physicians relative to the general US population. Arch Intern Med. 2012 Oct 8;172(18):1377-85. doi: 10.1001/archinternmed.2012.3199. PMID: 22911330.]
